# Negotiating care in organizational borderlands: a grounded theory of inter-organizational collaboration in coordination of care

**DOI:** 10.1186/s12913-024-11947-4

**Published:** 2024-11-20

**Authors:** Ann-Therese Hedqvist, Catharina Lindberg, Heidi Hagerman, Ann Svensson, Mirjam Ekstedt

**Affiliations:** 1 Department of Health and Caring Sciences, Linnaeus University, Kalmar/Växjö, Sweden; 2 Ambulance Services, Region Kalmar County, Västervik, Sweden; 3 School of Business, Economics, and IT, Division of Informatics, University West, Trollhättan, Sweden; 4 Department of Learning, Informatics, Management and Ethics, LIME, Karolinska Institutet, Stockholm, Sweden

**Keywords:** Complex care needs, Constructivist grounded theory, Care coordination, Integrated care, Inter-organizational collaboration

## Abstract

**Background:**

Although coordination of care and integrated care models aim to enhance patient satisfaction and perceived care quality, evidence regarding their practical implementation remains scarce. Understanding the nuances of collaboration across care providers to achieve effective coordination of care is imperative for seamless care integration. The aim of this study was to construct a grounded theory of how inter-organizational collaboration is performed to support coordination of care for patients with complex care needs.

**Methods:**

A qualitative design with a constructivist grounded theory approach was applied. In total, 86 participants with diverse backgrounds were recruited across multiple care settings, including hospitals, ambulance services, primary care centers, municipal home healthcare and home care services. The grounded theory was developed iteratively, based on a combination of observations and interviews, and using constant comparative analysis.

**Results:**

Coordination of care, a complex process that occurs across interconnected healthcare organizations, is manifested as “Negotiating care in organizational borderlands.” Care coordination evolves through a spectrum of inter-organizational collaboration, ranging from “Dividing care by disease-specific expertise” to “Establishing paths for collaboration” and ultimately “Co-constructing a comprehensive whole.” These categories highlight the challenges of coordinating care across both professional and organizational boundaries. In the multifaceted healthcare landscape, effective care coordination occurs when healthcare professionals actively bridge the divides, leveraging their collective expertise. Importantly, organizational boundaries may serve a purpose and should not be dissolved to facilitate effective care coordination.

**Conclusions:**

The key to effective care coordination lies in robust inter-organizational collaboration. Even when patients receive integrated care, healthcare professionals may have fragmented roles. This research emphasizes the importance of clearly defined lines of accountability, reinforcing mutual responsibility and facilitating bridging of professional and organizational boundaries. Healthcare professionals and policymakers can use these insights to effectively utilize inter-organizational collaboration in supporting care coordination for patients with complex care needs.

**Supplementary Information:**

The online version contains supplementary material available at 10.1186/s12913-024-11947-4.

## Introduction

Healthcare systems worldwide are facing challenges in providing care for the increasing number of people with chronic illness and multimorbidity, requiring healthcare and social care from multiple care providers [1, 2]. Advances in technology and specialized care approaches have extended life expectancy, even for those with multiple chronic conditions [3]. Concurrently, healthcare reforms have shifted care delivery, resulting in shortened hospital stays and increased reliance on outpatient and home care [4]. This shift has led to a specialized, decentralized, and highly professionalized healthcare system [5] requiring patients to navigate care from multiple experts. Such specialization often leads to care fragmentation across various providers, sometimes creating silos that hinder coordination between care providers.

“Complex care needs” refers to the multidimensional needs arising from chronic conditions, physical or cognitive disabilities, mental health issues, and social determinants of health [1, 2]. Individuals with such health challenges often have to navigate a complicated healthcare system, figuring out when and where to seek assistance [6]. The risk of information gaps and fragmented care makes individuals with complex care needs particularly vulnerable [7]. Without a cohesive care plan, care may be omitted and services duplicated—not only wasting valuable organizational resources but also causing unnecessary discomfort for patients [8, 9]. Inter-organizational collaboration enables professionals from various disciplines to actively work together, share knowledge, and coordinate strategies for seamless care integration. The increasing complexity of care needs highlights the urgent need for efficient collaboration models [10–12].

## Background

The healthcare system is characterized as a complex, adaptive system, too intricate for any single individual to comprehend fully [13]. Although this multifaceted structure offers numerous advantages, it also presents challenges [14]. Because care providers operate under various laws and regulations, each addressing a specific aspect of the care trajectory, unforeseen gaps in care coordination may occur [7, 15]. Given the complexity, no single entity can provide the full range of competencies required to address complex care needs. This calls for healthcare and social care experts—henceforth referred to as healthcare professionals—working together across various healthcare domains, including in-hospital care, ambulance care, primary care centers, municipal home healthcare, and home care services.

Inter-organizational collaboration is defined as a mutually beneficial process where organizations work together towards a shared goal. It involves the joint development of structures in which decisions are made collectively, resources are shared, and mutual authority and accountability are exercised [16]. Such collaboration is characterized by a common underlying commitment to achieving benefits that would not be possible if each organization worked independently [17]. Coordination refers to reduction in duplication of effort, reduction in gaps of services, and sharing of knowledge and skills [18, 19]. The degree to which organizations are coordinated is a key to the success or failure of collaborations [20, 21]. Previous studies [8, 22–27] have demonstrated that collaboration is central in primary care during care transitions and coordinated discharge planning for individuals with complex care needs. Research on coordination of care underscores the importance of comprehensive patient knowledge, strong inter-provider relationships, and the capacity to bridge healthcare system gaps for safety and effectiveness [28, 29].

Transition coordinators are widely recognized for their important role in assisting with navigation of intricate healthcare pathways. Integrated care models aim to overcome the issue of fragmented healthcare and social care by offering a seamless continuum of coordinated services for individuals with chronic illness [30, 31]. The effectiveness of these models stems from their ability to aid navigation in the intersectoral landscape of healthcare, facilitating connections between various care providers [32–34]. For instance, the Transitional Care Model involves use of transitional care nurses for discharge planning and at-home follow-ups. The Transitional Care Model addresses immediate patient needs post-discharge and has been shown to effectively reduce readmissions [35, 36].

Our study focuses on the social processes, interactions, relationships, and communication patterns across organizations through which healthcare professionals collaborate to address the comprehensive needs of individuals dependent on the healthcare system, particularly patients with chronic or long-term needs [37]. Although integrated care models have benefits, they also introduce increased interdependencies between care providers across healthcare and social care sectors [38], requiring more collaboration for care to function effectively. For patients and their families, truly integrated care is realized when their needs and preferences are at the forefront of care delivery [39], fostering an experience of seamless care.

Worldwide, there is a movement towards enhancing integrated care to better align healthcare with patient needs [40, 41]. Many developed countries, including England, Canada, and Sweden, have recently reformed their systems, emphasizing enhanced primary care and coordinating services for older people and those with chronic illness [4]. International guidelines advocate community-based, multidisciplinary care and emphasize the need for designated care coordinators [38]. Realizing these ambitions of integrated care necessitates an interconnected healthcare system and strengthened inter-organizational collaboration [42, 43]. Despite global strides taken to integrate healthcare and social care services, implementation of integrated care into practice remains unsuccessful [44]. This underscores the importance of understanding how inter-organizational collaboration can effectively deliver integrated care across healthcare and social care systems. The ongoing reforms aimed at shifting towards more home-based care provide a unique opportunity to study how transitions towards more effective cross-organizational coordination of care can be managed through collaboration. Therefore, the aim of this study was to construct a grounded theory of how inter-organizational collaboration is performed to support coordination of care for patients with complex care needs.

## Methods

### Design

An ethnographic approach [45] with constructivist grounded theory methodology [46] was employed in this study. This combination allows for a rich understanding grounded in the real-world experiences of the participants while generating theory closely linked to the data. Inter-organizational collaboration and coordination of care involves multiple stakeholders, diverse settings, and dynamic interactions. The flexible and iterative nature of the ethnography and constructivist grounded theory allows methods to adapt to the specific context of the study. Such flexibility facilitates a comprehensive exploration of the processes involved in coordinating care across care provider boundaries for patients with complex care needs.

### Trustworthiness

To ensure the trustworthiness of this study, we adhered to the four quality constructs: credibility, originality, resonance, and usefulness [46]. The constructivist grounded theory approach acknowledges that researchers bring their own perspectives to the research process, which can shape and enhance a study. Reflexivity was central to ensuring the trustworthiness of the research while respecting and valuing subjectivity [47]. Personal reflexivity involved continuous self-reflection by the research team, to assess how their backgrounds influenced the research process and ensure that professional preconceptions did not overshadow the participants’ real-world experiences. The majority of the research team members were registered nurses and a non-nursing team member added valuable external viewpoints that enriched the interpretations in the study. Interpersonal reflexivity was fostered through regular peer debriefings and team discussions, allowing diverse perspectives to challenge assumptions and enhance the credibility of the analysis. Methodological reflexivity involved critically examining the implications of data collection and analysis strategies [46, 47]. Contextual reflexivity accounted for the broader healthcare environment and organizational cultures that influenced participants’ interactions and the research context, ensuring that the findings were grounded in real-world practices. Resonance was strengthened through interprofessional seminars, where healthcare professionals reviewed and provided feedback on the findings, challenging and confirming their practical relevance for real-world application [46]. Direct participant quotations supported the authenticity of the analysis. Originality stemmed from the multidisciplinary perspective of inter-organizational collaboration, providing new insights into care coordination. Usefulness was demonstrated through the robust methodology and comprehensive data collection, with potential applicability in other settings, considering contextual variations. The report of the study followed the Consolidated Criteria for Reporting Qualitative Studies (COREQ) [48] (Appendix S1), ensuring thorough and transparent reporting.

### Study setting

Sweden has a decentralized healthcare system with some parts managed and run by regions and others by municipalities [49]. Regions oversee primary, secondary, and specialist care. Primary care is further divided into healthcare (i.e., healthcare centers) and rehabilitation. The municipalities are responsible for home healthcare and social care, including services like elderly care, nursing homes, and home care services. The regulatory structure separates the two sectors, with the Health and Medical Services Act governing healthcare, and the Social Services Act governing social care [50, 51].

Although the national government establishes the overarching political direction for healthcare and social services and enacts laws and regulations, self-governance is integral to the operations of regions and municipalities. This autonomy enables regions and municipalities to design services adapted to the specific needs of the local population. Although self-governance injects a level of flexibility and diversity into the system, it can also create complications. Notably, there is a potential for variability in the accessibility and quality of healthcare across different regions [49]. One manifestation of self-governance is the freedom that each region possesses in choosing its preferred documentation system, occasionally resulting in system incompatibilities between regions. The region where the study was conducted is one of the counties with the highest proportion of older citizens in Sweden. This creates a need for efficient and accessible healthcare services. The region includes urban, rural, and sparsely populated areas. Such diversity introduces a range of challenges to and opportunities for healthcare delivery. Collaboration between the region and its municipalities is well-established. These characteristics, along with the region’s collaborative infrastructure, made it an ideal setting for this study, offering the potential for valuable insights into real-world practices of inter-organizational collaboration and coordination of care.

### Recruitment of participants

To capture a range of demographic and geographic variations that could influence organizational conditions and cultures within the chosen region, a selection of four municipalities, three primary care centers, four ambulance stations, and three regional hospitals, was made. The ambition was to ensure that participants represented a wide range of professional roles across different care providers involved in inter-organizational collaboration, to capture the social processes, interactions, and communication patterns of care coordination for patients with complex care needs. After ethical approval was obtained, the top manager of each care provider received written and verbal information about the purpose of the study. Informed consent was obtained to contact the respective heads of department, who then decided whether to allow or refuse access to sampling in their units. Access was granted to all but one unit, which cited increased workloads and isolation due to the COVID-19 pandemic as reasons for declining participation. Appointed contact persons at the hospital departments, primary care centers, ambulance services, and municipal home healthcare conveyed contact with eligible staff to the research team and gave access for observations.

Purposive sampling methods were used initially to select the participants most likely to provide experiences on inter-organizational collaboration, enhancing usability. This included healthcare professionals from diverse disciplines and care settings, covering different organizational structures and conditions for collaboration and coordination of care. Theoretical sampling followed during the analysis process, driven by emerging findings. As data collection and analysis progressed, a need for additional potential participants to fill conceptual gaps was identified. Sampling, data collection, and analysis were performed in iterative cycles until theoretical saturation was achieved [46].

To ensure a multidisciplinary perspective on inter-organizational collaboration, 86 healthcare professionals (70 women and 16 men) were recruited from domains including in-hospital care, ambulance services, primary care centers, municipal home healthcare, and home care services (Table 1). Each of these professionals contributed to the care of individuals with complex care needs. To maintain clarity in the reporting of the findings and ensure confidentiality, each quotation from a participant is accompanied by an identifier with a number and the participant’s profession and organization, formatted as “(P[number], [profession], [organization]).” This approach ensures that insights remain contextually grounded while individual identities are protected.

**Table 1:** Summary of study participants

		**Interviews**		**Participant observations**	
		Semi-structured interviews *n* = 57	Informal interviews *n* = 15	Individual observations *n* = 13	Meeting observations *n* = 10
	**n**	**n**	**n**	**n**	**n**
Medical and geriatric hospital ward					
Registered nurse	5	1	4	4	3
Care coordinator	4	4	2	2	8
Physician	5	3	2		1
Ambulance service					
Ambulance nurse	10	10			
Primary care center					
Care coordinator	7	2	2	2	7
Physician	6	4	2	2	
Municipal home healthcare					
Occupational therapist	8	4			7
Physiotherapist	7	5			6
Registered nurse	14	12			3
Care coordinator	7	2	3	3	6
Municipal home care services					
Social service officer	5	2			8
Assistant nurse	6	6			
First-line manager	2	2			
**Participants total**	**86**	**57**	**15**	**13**	**49**

### Data collection methods

In an interpretive approach [46], data are systematically and concurrently collected and undergo constant comparative analysis. Here, data were collected between June 2020 and April 2022, utilizing a combination of convergent data collection techniques for data triangulation [46], promoting credibility by ensuring a comprehensive understanding of inter-organizational collaboration and care coordination. The data collection process included participant observations (shadowing), meeting observations, semi-structured interviews, and informal interviews with healthcare and social care professionals in connection with observations (Table 1). By using interviews as a complementary method, we were able to deepen our understanding of social processes and interactions that were difficult to capture through mere observations. Interviews provided insights into participants’ thoughts, reasons for decisions, unspoken assumptions, interactions, and cognitive work.

### Observations

Observations were conducted to capture inter-organizational collaboration and care coordination in real-world settings, providing authentic data that reflected organizational structures, procedures, and behaviors of healthcare professionals. This approach allowed for the capturing of non-verbal communication, body language, and social dynamics that would be missed in interviews, enabling a deeper understanding of the social processes and interactions.

The observation process involved several distinct yet interrelated steps: growing familiar with the research domain, shadowing healthcare professionals, and conducting structured observations [52, 53]. The primary focus was on capturing the dynamics of inter-organizational collaboration, information exchange, and the management of care transitions, including discharge planning, inter-organizational meetings, and patient handovers. In individual observations, we focused on real-time interactions between healthcare professionals, particularly their roles, interactions, communication patterns, and how they coordinated care for patients.

Observations were conducted by the first author, who had a background as a registered nurse, ensuring an informed perspective throughout the process. Initial participant observations discerned key activities, roles, and disciplines for targeted data collection. Insights gained from these initial observations were used to develop an observation guide. This guide outlined specific areas of interest, such as information exchange during discharge planning, and facilitated systematic documentation of interactions.

Subsequently, healthcare professionals were shadowed, focusing on daily workflows and interactions, with a basis in the observation guide. Structured observations were also conducted during various team meetings and care coordination meetings, attended exclusively by multidisciplinary teams of professionals, as well as of care planning sessions and patient discharge conversations involving patients and their families. Observations were conducted in multiple healthcare environments, including hospital wards (20 sessions), primary care centers (six sessions), and municipal home care settings (ten sessions). Each session was limited to a maximum of four hours, to promote focus and minimize observer fatigue. In total, 13 individual observations of healthcare professionals and ten inter-organizational meetings were conducted, resulting in a total of 93 h of observation.

To clarify any questions emerging from observations and enhance our contextual understanding, informal interviews were conducted linked to the observed events. Concurrently, real-time field notes were taken, capturing significant details and observations. These notes were revisited later to ensure precision and thoroughness. Overall, this structured approach ensured a comprehensive understanding of the dynamics at play during inter-organizational collaboration.

### Interviews

The combination of observation and interviews with key individuals, as detailed in Table 1, enriched the study’s originality by providing unique insights into the processes observed. Semi-structured interviews provided greater depth by yielding information on the observed interactions and allowing participants to articulate their reasoning, assumptions, and decision-making. This approach contributed to the practical relevance and usefulness of the study’s findings.

The initial interview guide (Appendix S2) featured open-ended questions such as “Can you describe a successful collaboration regarding a patient?” and “What made it successful?” The interview guide was refined as initial data were analyzed, in line with constructivist grounded theory [46], to integrate emerging insights and questions. During interviews, probing was employed to elicit detailed responses from participants.

Due to the challenges posed by the COVID-19 pandemic, interviews were scheduled and conducted in accordance with participants’ preferences, with in-person, telephone, or virtual meetings via platforms like Skype or Zoom being offered [54]. This adaptability ensured uninterrupted data collection while respecting pandemic protocols and restrictions. Interviews ranged in length from 32 to 75 min. All interviews were recorded and transcribed verbatim, capturing the intricacies of the participants' perspectives. Transcription services were used due to the large volume of material collected. However, to strengthen trustworthiness, the research team thoroughly reviewed each transcript and engaged with the data throughout the analysis process.

### Data analysis

**Fig. 1:**
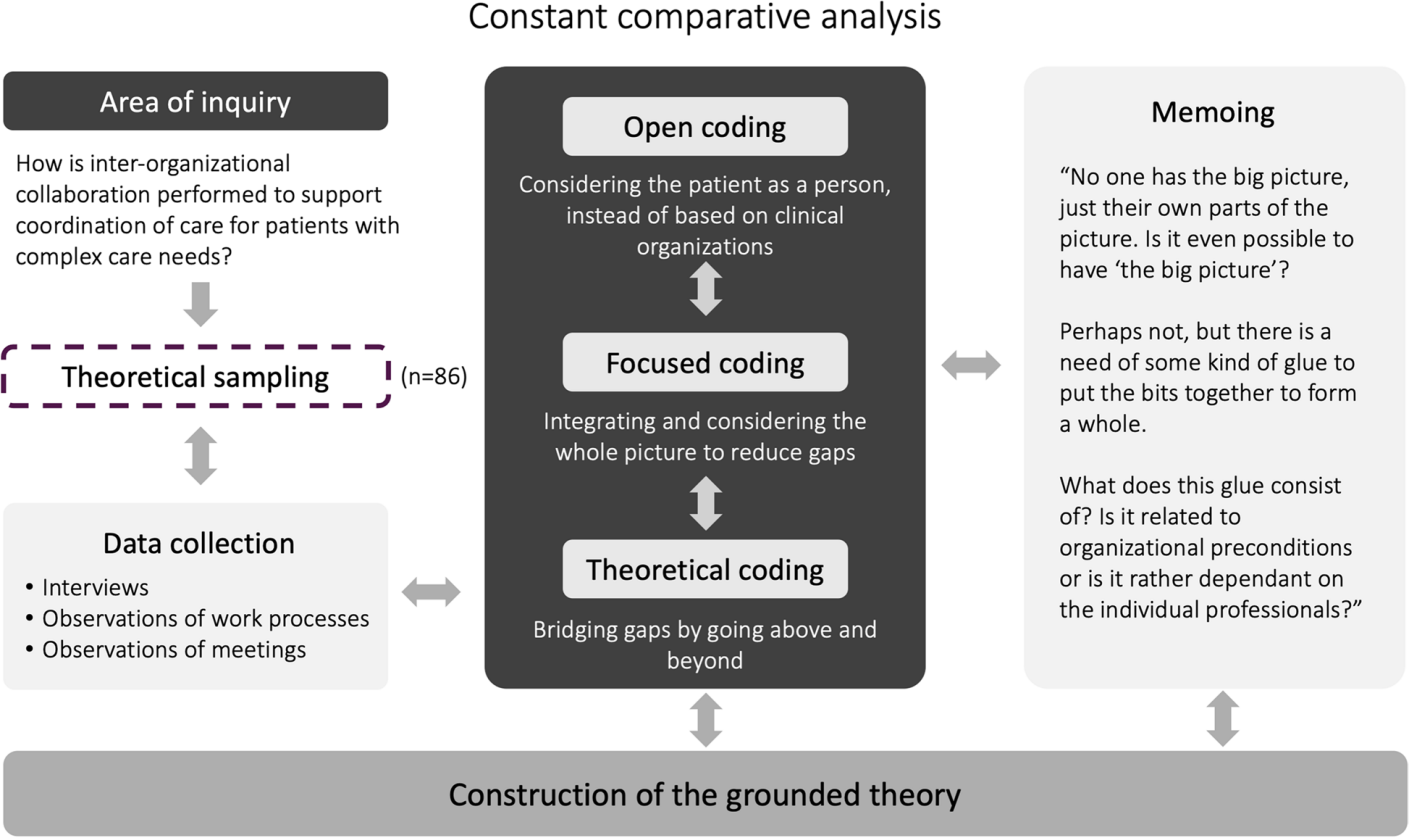
Illustration of the analysis process with examples from coding and memoing

Data analysis incorporated open coding, focused coding, theoretical coding, and ongoing memo writing [46] (see examples in Fig. 1). Credibility was strengthened through immersion in the data, enabling a deep understanding of the real-world processes of inter-organizational collaboration and care coordination.

Analysis began after the initial observations and interviews, with field notes and transcripts being reviewed to create an overview of the collected data, as multiple data sources were analyzed concurrently. Next, our attention shifted from fieldwork to in-depth data analysis [55], starting with the delineation of segments. A segment was defined as any portion of data where a distinct action, social process, or interaction occurred, in relation to inter-organizational collaboration, with boundaries drawn based on shifts in focus, such as transitions between different aspects of care coordination [46]. During open coding, each segment was assigned a descriptive code, typically in the gerund form, to depict actions and themes, addressing the query “What action is happening here?” In vivo codes were used whenever applicable. Microsoft Excel™ was utilized to effectively manage the vast amounts of data. As connections between actions, processes, or interactions emerged, significant codes were deliberated upon to clarify their importance and corroborate them with the extant data. Throughout this process, we intermittently returned to the field, procuring supplementary data via interviews and observations, ensuring that analytical queries were addressed, and any conceptual gaps were filled [46].

After identifying the initial codes that appeared most frequently or held significant relevance, focused coding was performed, facilitating the synthesis and sorting of vast amounts of data [55]. These focused codes underwent thorough examination, so we could determine which best explained the observed phenomena. The codes were then contrasted and organized into tentative categories. The first author initiated the analysis of these tentative categories through focused coding, after which the entire author group deliberated on them. Trustworthiness was reinforced by the authors cyclically returning to the primary data for verification. This iterative process ensured that emerging themes and patterns were identified and refined throughout the study. Continuous data comparison aided refinement of the categories, with the ultimate aim of consolidating them into fewer, highly meaningful categories, as suggested by Charmaz [46].

**Fig. 2:**
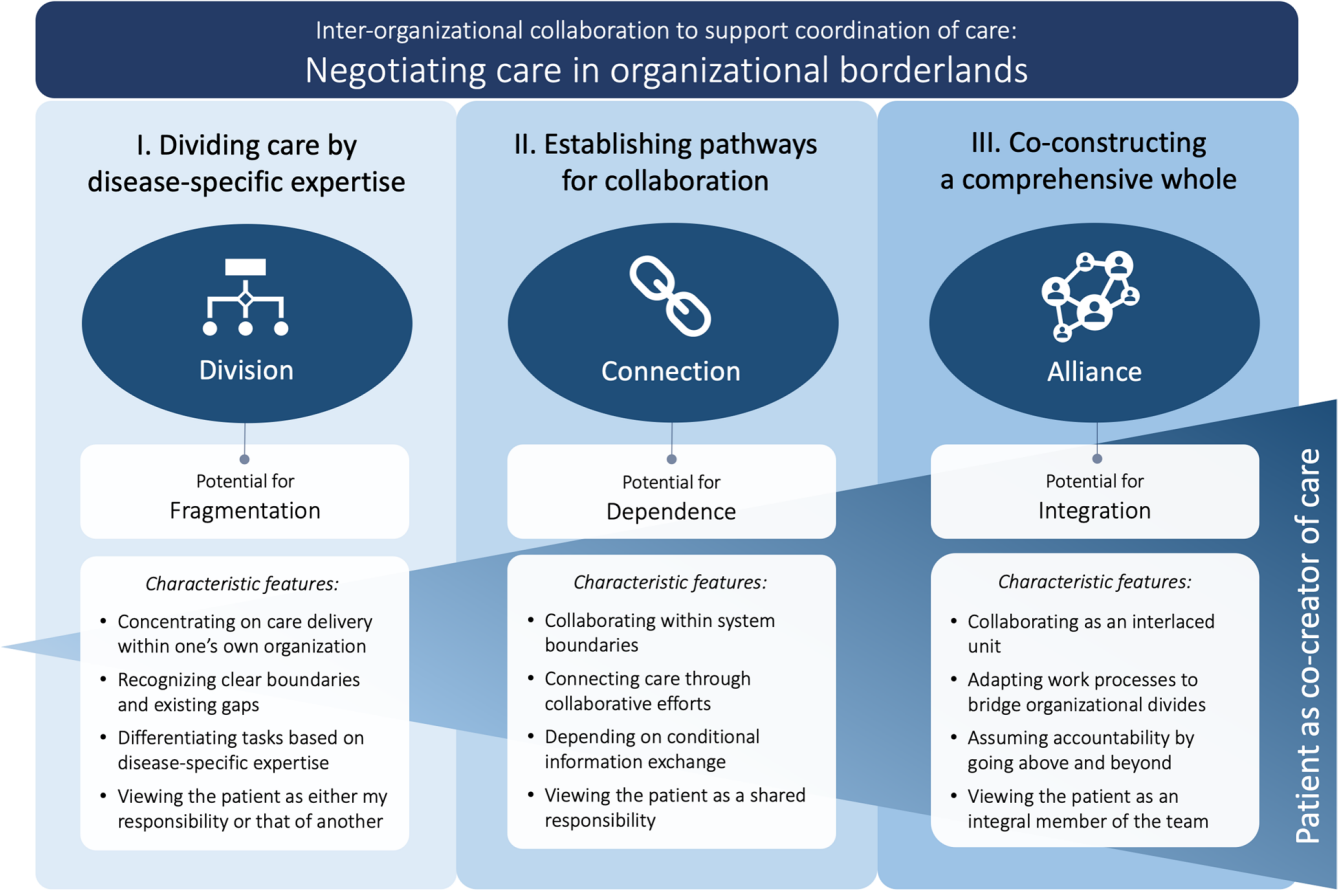
A grounded theory of inter-organizational collaboration to support coordination of care. This figure illustrates the inter-organizational collaboration, showing levels of care integration

Next, theoretical coding was employed to delineate the potential relationships between the developed categories, weaving the segmented elements from the focused coding into a cohesive and comprehensible premise, culminating in a theory. Throughout this conceptual evolution, a methodical structure was consistently maintained, continually juxtaposing data, codes, categories, and emerging concepts. Once no further relationships between the categories were identified, the categories and their respective subcategories were integrated into a core category and finally constructed into a grounded theory (Fig. 2) to address the aim of the study.

### Ethical considerations

Prior to data collection, both verbal and written informed consent procedures were established to provide participants with detailed study information, address any queries, and obtain their agreement to participate, with the possibility to withdraw at any point. No sensitive personal information was collected. Continuous reflection on the observer’s role and influence promoted research sensitivity and integrity while safeguarding participant wellbeing. This study was approved by the Swedish Ethical Review Authority (registration number 2020–01219) and complied with the ethical guidelines of the Helsinki Declaration [56].

### Findings

The grounded theory of inter-organizational collaboration to support coordination of care is manifested as “Negotiating care in organizational borderlands.” This theory is constructed by three distinct yet interconnected categories: “Dividing care by disease-specific expertise,” “Establishing paths for collaboration,” and “Co-constructing a comprehensive whole.” The categories reflect the potential for fragmentation, dependence, and integration within care coordination. Although each category represents a stage along a spectrum, they are not mutually exclusive. In practice, elements from each category often coexist, with the care coordination process incorporating aspects of division, connection, and alliance simultaneously, depending on the context (Fig. 2).

### Negotiating care in organizational borderlands

“Negotiating care in organizational borderlands” captures the delicate balance of harmonizing professional roles, expertise, and patient needs within the healthcare landscape. Inter-organizational collaboration is inherently complex, unfolding within interconnected healthcare organizations, and—notably—in the organizational borderlands that separate and define them. These borderlands, where various professional territories intersect, require active negotiation to ensure seamless integration of care for patients with complex care needs. Depending on the nature of the patient's situation and the complexity of care, collaboration may manifest as division, connection, or alliance.

Navigating these borderlands begins with defining each profession's domains or areas of expertise. Observations during inter-organizational meetings revealed how healthcare professionals actively negotiated their roles and responsibilities to coordinate care effectively. Determining organizational limits and responsibilities enabled professionals to coordinate care and contribute effectively to collaboration. A clear understanding of the borders of professional domains is essential to promote accountability. Once domains are defined, professionals must identify gaps or overlaps in care. The negotiation process here involves the coordination of care to fill these gaps.

The progression from division via connection to alliance on the spectrum of inter-organizational collaboration (Fig. 2) is driven by negotiation processes among healthcare professionals. At *division*, physicians and specialists within individual organizations or specialties are primarily responsible for setting up care based on disease-specific expertise, a practice often reinforced by organizational structures and legislative frameworks. These boundaries can inadvertently inhibit negotiation processes, leading to compartmentalized care—the “silo mentality.” This constrained perspective, held by professionals focused on their specialized roles, risks eroding the integrative view necessary for integration of care and highlights the complexities of negotiating care in organizational borderlands. However, moving along the spectrum to *connection*, pathways for collaboration across boundaries are constructed. As observed during care transitions from hospital to home, active negotiations between healthcare professionals from different professions and organizations become paramount. Such negotiations involve physicians, nurses, care coordinators, rehabilitation experts, and social service officers working together to bridge gaps in care and harness collective knowledge. Lastly, in *alliance*, the negotiations culminate in a co-construction process, where healthcare professionals, together with the patient and family, form an interlaced unit with potential for integration—contributing to the experience of seamless care.

### Dividing care by disease-specific expertise

The practice of dividing care based on disease-specific expertise presents itself as both an advantage and a drawback. Although this approach aims to ensure that patients benefit from focused, specialized attention tailored to their specific health conditions, it also harbors potential pitfalls. It may result in discontinuities in care, exacerbated by the diverse cultures of various healthcare professions and the existence of informational silos. Observations and interviews with healthcare professionals highlighted that stereotyping among professionals from different disciplines in some cases was a barrier to collaboration, as assumptions about roles and expertise obstructed open communication and mutual understanding. For example, ambulance nurses were occasionally perceived as focusing solely on acute situations, which led hospital staff to overlook their insights regarding the broader context of patient care.

This compartmentalized approach to healthcare involved each specialty focusing predominantly on its expertise-related tasks. Observations of patient handovers in hospitals highlighted that healthcare professionals often prioritized their immediate responsibilities, which limited their engagement with the broader, long-term needs of each patient. Due to the time-constrained and resource-limited nature of specialist care, deeper exploration into associated issues or future concerns, such as long-term self-management or follow-ups, was typically referred to professionals in primary care or municipal home healthcare. Understanding the insights and actions of colleagues across organizational boundaries was experienced as essential by many, especially primary care physicians and municipal home care staff, who often felt disconnected from hospital-based care processes. However, contrasting understandings among healthcare professionals across these settings frequently revealed a disconnect, as illustrated by the following quotations. In the hospital setting, the focus is often on addressing specific medical issues in isolation:

*Healthcare is organized so that you treat one thing at a time…we’ve only seen a small part of the problem, but maybe haven’t done a full analysis because there isn’t time for that. We have to be satisfied that the patient survived and could be discharged. (P12, physician, medical hospital ward)*

On the other hand, a primary care physician expressed frustration over the apparent one-directional flow of information, stating that:

*There's a noticeable lopsidedness in communication... I can't recall instances when a hospital physician reached out directly. Communication is typically routed through referrals. There's a sense that specialists may not be thoroughly reviewing our records, which sometimes leads to redundant requests for tests or procedures we've recently conducted. Like—can you investigate this? Yeah, we already did that, two months ago. (P22, physician, primary care)*

Healthcare professionals emphasized the diversity of their professional viewpoints, which occasionally resulted in misunderstandings. Such misunderstandings could materialize as practical problems, including missed or duplicated testing and unnecessary examinations. The unique cultures of various professions often acted as communication obstacles, comparable to “speaking in different languages.” These obstacles posed a challenge to efficient information exchange and sharing of data. Although specialized knowledge within healthcare was considered an invaluable asset, the varying professional cultures could impede effective communication.

A troubling consequence of this fragmented approach was the emergence of informational silos, as observed during shadowing of healthcare professionals in hospital and municipal home healthcare settings. In many cases, healthcare professionals lacked access to complete patient information, particularly during care transitions. Such silos could mean that vital patient information is obscured or inaccessible, which was especially problematic in urgent prehospital situations. Ambulance nurses, for instance, often found themselves “operating in the dark,” lacking full background details and were forced to base decisions solely on a patient's present condition. Siloed information systems also made access to comprehensive patient information difficult, creating barriers to mutual understanding. Illustrating this fragmented approach, a registered nurse in municipal home healthcare described their experience:

*I enter the patient information...to see what he needs help with. Then I have to access another system to see background information on what illnesses he has and yet another system to see his medications. And in another system, I see which physician and healthcare center he has... Someone else enters what contact persons he has... There are a lot of different systems I have to open to write the records. (P13, registered nurse, municipal home healthcare)*

The level of integration in inter-organizational collaboration is often revealed through the nature of interactions between healthcare professionals and the degree of shared responsibility in patient care. This was identified by observing communication patterns and the extent to which providers from different organizations or specialties actively coordinated care. A lower level of integration tended to foster underlying tensions, where collaboration was fragmented or incomplete. In such instances, a culture of "us versus them" often emerged among healthcare professionals, reflecting both professional and organizational cultural divides. Responsibility for the patient was then seen as belonging either to oneself or to others—described as "someone else's" responsibility. During shadowing and interviews, we observed that when boundaries between roles and organizations were unclear, or responsibilities were ambiguous, it could lead to miscommunication and overlooked tasks. Healthcare professionals might trust that someone else would take on certain responsibilities, as described by a registered nurse in municipal home healthcare:

*…it’s quite easy to overlook some things, especially when so many people are involved…it’s easy to miss something or to think that someone else will do it instead. (P5, registered nurse, municipal home healthcare)*

Observations and interviews revealed the integral role of healthcare professionals such as assistant nurses in home care, who interact with patients in daily life, particularly in maintaining safety after hospital discharge. Through their close and regular interactions with patients, these professionals are provided with unique insights into patients’ conditions and needs. However, both the observed discharge processes and interviews with different healthcare professionals indicated that the insights of assistant nurses were often underappreciated and not consistently integrated into inter-organizational care planning meetings. During coordination meetings, it was noted that information from social service officers and care coordinators often took precedence, even though it did not always capture the full extent of a patient’s situation.

### Establishing pathways for collaboration

Creating effective pathways for collaboration across organizational boundaries helps to bridge fragmented parts of the healthcare system. As healthcare professionals coordinate care in this complex landscape, collaboration goes beyond simply exchanging information—it requires developing coordinated strategies that align care efforts across different organizations and specialties. For example, in cases of patient discharge, healthcare professionals from hospitals, primary care, and municipal home care must not only share information but also work together to ensure continuity of care through coordinated follow-up, medication management, and home care services. This approach ensures that each provider understands their role in the broader care plan, helping to reduce gaps in care and prevent duplication of efforts.

Shadowing and interviews with healthcare professionals revealed that they often described their roles as puzzle pieces—each essential to completing the overall picture of comprehensive care. This analogy captures the interdependencies observed in daily practice, where inter-organizational collaboration is needed to bridge the gaps between organizations that may otherwise hinder effective coordination of care. The expression "our patient," used recurringly by healthcare professionals during interviews, highlights the aspiration for shared responsibility, reflecting the desire to work together across professional and organizational boundaries to provide cohesive care.

A mutual understanding among healthcare professionals ensures that care remains cohesive, bridging both professional and organizational boundaries. This mutual understanding involves not only a shared interpretation of the patient’s situation but also a clear knowledge of each other’s roles and responsibilities within the care team. It is further supported by effective coordination and consistent communication. A central entity, often the registered nurse, was recognized as vital in navigating the intricate web of healthcare, ensuring seamless communication and collaboration across organizational borderlands. However, several healthcare professionals viewed themselves as this “spider in the web” or central coordinator of patient care. Both registered nurses and social service officers considered their role essential in connecting various aspects of care:

*The nurse essentially plays this role. We collaborate with everyone, positioning ourselves at the center of coordination, thus becoming the “spider in the web.”(P25, registered nurse, municipal home healthcare)*

*As social service officers, our role is crucial. We are the “spider in the web,” acting as a central hub, connecting various services and providers. (P24, social service officer, social services)*

Still, there was an underlying sentiment that no single role had a complete overview of the entire process. Having such a centralized figure may be unattainable within the current system:

*There often seems to be no one with a holistic overview. We might have occupational therapists, physiotherapists, nurses, or social service officers, but I think it often comes down to individual initiative... There's no clear-cut role for this. (P4, occupational therapist, municipal home healthcare)*

In accordance with how healthcare professionals described the ideal process, centralized coordination was intended to streamline care plans across different care providers to ensure seamless care for patients. Such a process typically involved designated care coordinators working across primary care, municipal home healthcare, and hospital wards. Although registered nurses were responsible for overseeing certain aspects of care coordination, this structure was often viewed as insufficient for providing a holistic view of the patient's entire care journey. Healthcare professionals suggested in interviews that a specialized single point of contact could instead be used to enhance collaboration and improve coordination across different healthcare domains. The envisioned role would allow for a more comprehensive understanding of the patient's needs and care trajectory. However, it was also recognized that this role would come with significant challenges and responsibilities, especially considering the complexities of inter-organizational collaboration.

Due to organizational boundaries, healthcare professionals were found to utilize diverse communication channels, often dictated by the urgency of the situation and what was most convenient for the involved parties. In the absence of integrated information systems, healthcare professionals relied heavily on selective information sharing. For instance, care coordinators and registered nurses spanning hospital, primary, and municipal home healthcare settings crafted their own channels to facilitate the necessary information flow.

Interviews and observations revealed that intra-organizational communication, for instance between municipal home healthcare nurses and assistant nurses, typically occurred through direct face-to-face interactions or phone calls. In contrast, inter-organizational exchanges were largely conducted using digital tools, prioritizing efficiency and timeliness. However, participants frequently emphasized the importance of in-person, synchronous interactions in enhancing communication. Well-established professional relationships and face-to-face interactions were found to significantly improve the quality of communication, leading to stronger collaborations. This was especially important in complex cases, where face-to-face communication minimized misunderstandings and promoted clarity. In essence, communication stands as the foundational pillar in successful inter-organizational collaboration. As one municipal home healthcare physiotherapist succinctly put it:

*...good communication is key. Misunderstandings often arise when there's a lack of dialogue. So... yeah, it’s usually not more difficult than that. (P2, physiotherapist, municipal home healthcare)*

### Co-constructing a comprehensive whole

This category represents a position on the spectrum of inter-organizational collaboration, highlighting the role of alliances in coordination of care. Alliances reflect a high level of collaboration, where collective problem-solving and sustained interprofessional communication help overcome organizational barriers. Additionally, they promote individualized care by actively involving patients and their families in decision-making, harnessing their expertise for a more inclusive approach to healthcare. The relational and ethical dimensions of care were frequently emphasized by participants, who underscored the moral responsibility that healthcare professionals have when working across organizational boundaries. These ethical considerations, such as the need to respect patient autonomy and prioritize person-centered care, often guided decision-making and strengthened the collaborative process. As healthcare professionals and patients united in shared knowledge and decision-making, various aspects of care were seamlessly integrated. Although formal responsibility for patient care may still reside within each organization’s legal framework, in practice, collaborations foster a sense of shared responsibility across organizational boundaries. Healthcare professionals from diverse organizations work together, coordinating their efforts to align with patient needs and preferences. However, formal structures for sharing responsibility across different jurisdictions may vary depending on local policies and agreements. Participants in the study described these joint efforts as feeling like "working as one" and "experiencing flow," capturing the sense of alignment and seamless collaboration they aimed to achieve.

The core premise revolves around tailoring healthcare organizations to meet patient-specific needs, rather than requiring patients to navigate rigid systems. This perspective shifts the focus from individual professional duties to a unified approach, softening the boundaries between organizations. A vital component of this integration involves healthcare professionals reaching out across organizational borderlands and sharing accountability by working together to problem-solve and collaborate. This process involves active dialogue, where professionals engage in meaning-making together to ensure a comprehensive understanding of patient needs. By “going the extra mile,” healthcare professionals ensure that care-related communication is not only clear but also a collaborative process that aligns their efforts and accountability.

In this category, where alliance is prominent, healthcare professionals were seen to frequently go beyond their designated roles to co-construct a comprehensive whole of the patient’s situation. This was manifested by healthcare professionals prioritizing the patient's wellbeing, ensuring that all their concerns were addressed. Instead of merely directing patients to other departments or services, they took proactive steps to guide them further:

*While it might not directly fall under my purview, I see it as my duty. In my world, it actually is. I believe in actively facilitating the connection, rather than merely advising patients to seek elsewhere. (P3, social service officer, social services)*

However, such commitment came with challenges. Assistant nurses highlighted the juggling act of managing multiple tasks and patient visits, especially where there were time or resource constraints. Such challenges intensified when new patients required immediate attention, affecting both assistant nurses and registered nurses. A municipal home healthcare nurse stated that:

*When something is missing or lacking, we nurses step in. We’re problem-solvers by nature, you tie yourself into knots to make sure the patient’s needs are met. (P1, registered nurse, municipal home healthcare)*

Inter-organizational team meetings were observed throughout the study as being integral to synchronizing patient care efforts across multiple care providers. These meetings fostered collaborative decision-making, allowing healthcare professionals to share their expertise and develop comprehensive care plans. Healthcare professionals from diverse disciplines—including in-hospital care, primary care, and municipal home healthcare—brought their specialized knowledge and specific organizational contexts to the table. These meetings were conducted with structured agendas that facilitated detailed discussions on patient cases, care plans, and challenges. Such inter-organizational meetings promoted collaborative decision-making by leveraging technology such as electronic health records, ensuring that all participants had access to up-to-date patient information. The primary focus of these sessions was to engage in productive dialogue that culminated in actionable care strategies, resulting in a jointly formulated care plan, leaving little room for things to be overlooked. With a shared mental model and understanding of the whole, seamless care—from the patient's point of view—was promoted, and the healthcare professionals felt safe in handing over the patient to one another. A registered nurse from municipal home healthcare described the essence of these meetings:

*We all gather together, and I get the answers I seek. At the same time, I might learn that this person will be getting a lot of home care...so I get an overall picture.(P18, registered nurse, municipal home healthcare)*

The holistic view was further strengthened by the ambition of involving the patient and their family as members of the team and active co-creators of care. The services were adapted to fit the patient's needs, as the healthcare professionals considered the patient to be a unique person with inherent resources, instead of focusing solely on the patient’s current illnesses or diagnoses. A care coordinator from a geriatric hospital ward reflected on this:

*While diagnoses are crucial, we see the elderly individual beyond them. We have it all, medical, surgical, infections, psychological...so many different diagnoses. You don’t become overly focused on the diagnosis itself, you just see this older person with their diagnoses... Recognizing their entirety, perhaps, enables us to truly understand and see them. (P29, care coordinator, geriatric hospital ward)*

In essence, "co-constructing a comprehensive whole" refers to the alliance of professional expertise and patient individuality, transcending organizational barriers to support coordination of care. It underscores the importance of adaptability, inter-organizational collaboration, and viewing the patient not as a passive recipient, but as an active collaborator in their own care.

## Discussion

This study examines the underlying structures of healthcare systems that either impede or enable collaboration. The findings revealed barriers to achieving seamless integration of care, such as strict legal frameworks, inconsistent information systems, and ambiguous roles and procedures. The study sheds light on the nuanced borderlands that define the interfaces between healthcare organizations. It identifies various levels of care integration, marking a transition from compartmentalized entities to cohesive alliances. The study contributes to a grounded theory on inter-organizational collaboration, challenging conventional organizational boundaries. The theory captures the dynamics of inter-organizational collaboration and the ethical imperative to co-construct care with patients and their families. We propose strategic interventions to reevaluate and potentially redesign organizational structures. These interventions are aimed at fostering a culture of collaboration and mutual understanding across diverse professional groups, helping to bridge existing gaps in care coordination.

### Intertwining roles and responsibilities in inter-organizational collaboration

Organizational borderlands are critical areas where healthcare activities converge and interact. These borderlands do not just define organizational boundaries; they are essential for negotiations that involve delineating professional territories, aligning goals, and bridging gaps in care. They also serve as fundamental components of healthcare systems and as vital communication interfaces [13]. Although normative dimensions such as mutual trust and shared goals have been increasingly recognized as essential for effective collaboration [32, 33, 57, 58], there is still a gap in understanding of how these elements function in practice. Unlike prior models, which focus on functional or logistical aspects [36, 59], our grounded theory also explores the relational and ethical dimensions of care coordination. This study illustrates that prioritizing communication, shared responsibility, and person-centered care can significantly enhance inter-organizational collaboration.

Effective collaboration across organizational borderlands requires clear understanding and recognition of each profession's and organization’s role. Healthcare professionals often describe their contributions as pieces of an intricate puzzle, collectively forming a unified picture. Phrases such as "experiencing flow" or "working as one" reflect the aspiration for an integrated approach, in line with what promotes well-coordinated efforts [32, 41, 60]. Each professional acts as a node within an interconnected network, where their expertise is amplified through collaboration with others [13]. No single node can support the system on its own. Rather, their interdependency requires coordination and strong relationships, especially when managing complex care needs [7, 15].

The findings reveal that to achieve seamless care, healthcare professionals often extend beyond their formal roles, crossing professional and organizational boundaries to adapt to individual patient needs, a result supported by Michgelsen et al. [61]. This adaptability points to a potential misalignment between rigid professional roles and the dynamic nature of patient care. Other studies align with this view [33, 62, 63], highlighting the importance of well-defined yet adaptable roles to create the necessary flexibility for meeting the diverse and evolving demands of patient care [41, 64].

The moral responsibility to act in the patient's best interest not only shaped decision-making but also strengthened bonds between professionals, creating a deeper level of trust across organizational boundaries. Trust and shared values enable professionals to move beyond task-based coordination toward sustained, meaningful collaboration. True integration is not achieved solely through efficient processes, but also by embracing shared responsibility. Reflecting Løgstrup’s concept of ethical demand [65], our study underscores the importance of interdependence and reciprocity, highlighting that each interaction can carry a profound moral responsibility that impacts patient care.

Our grounded theory also emphasizes shared accountability across care boundaries. In this context, accountability implies that each professional answers for their contributions to collective decisions [66]. Knowledge sharing and shared decision-making promote a mutual vision of care [32, 41, 67]. Regular coordination meetings and inclusive decision-making processes were found to be key for establishing shared goals and enhancing mutual accountability. However, to maintain sustainability, inclusivity must also be balanced with efficiency [61].

### Information sharing: risk or remedy for the integration of care?

In organizational borderlands, information sharing plays a vital yet challenging role, which varies depending on the achieved level of care integration. The grounded theory illustrates that in "division," barriers consist of limited access to information and a potential lack of interest in knowledge and information from other professions. In "alliance," despite increased information sharing, issues such as protective legislation and knowledge gaps between different care providers pose significant challenges [17, 36, 45]. A fully integrated system could facilitate a holistic view, connecting information across organizational boundaries. However, merely having access to data is not enough [68]; healthcare professionals must actively seek, share, and integrate patient information and knowledge to promote integration of care. This proactive engagement – despite systemic barriers – is driven by accountability and a commitment to patient care [57]. Effective communication within and between organizations is key to facilitating coordination of care, especially during care transitions [64]. The decentralized nature of contemporary healthcare, particularly with the growing emphasis on home-based care [4], necessitates the creation of multiple communication pathways.

Our findings showed that participants had a strong preference for direct, face-to-face interactions and dialogues over using technology for information sharing, especially during complex care transitions. Although previous literature highlights the importance of clinical information systems for decision support and information dissemination [66], this preference underscores the value of personal communication in complex situations where immediate feedback and real-time communication are vital. The healthcare professionals described these methods as more effective in preventing the miscommunication and delays often associated with asynchronous communication tools. The preference for these more "traditional" communication methods—such as in-person discussions—indicates a potential distrust or dissatisfaction with current technological systems, which participants felt were not always conducive to the nuanced and context-dependent nature of inter-organizational collaboration. Face-to-face communication fosters personal connections [69–71], enhancing trust and understanding among team members. The reliance on traditional methods suggests a need to improve clinical information systems to better align with healthcare professionals' workflows and communication preferences. Enhancing systems to become more synchronous, intuitive, and user-friendly could bridge the gap between technology and traditional methods, fostering more integrated and efficient care coordination [72]. In the movement towards integrated care, questions arise about the robustness of current healthcare systems. Specifically, one must consider whether these systems do in fact enable seamless information sharing and whether we are on the verge of redefining data management and knowledge dissemination in this transformative era of healthcare.

### Care coordinators: bridging the organizational divides?

The metaphorical representation of the care coordinator in the findings can be likened to “a spider in the web,” weaving an intricate web of multifaceted healthcare services. The significance of this role has been highlighted in earlier works [41]. Such a role functions as a singular, empathetic anchor, especially for older patients who seek guidance on their healthcare journey [73]. This essential function aligns seamlessly with strategies that cater to complex care needs [12] and is mandated in the Swedish legal framework, which emphasizes the importance of having a designated point of contact responsible for coordinating care. However, achievement of seamless care is shown to rest upon a triad: comprehensive patient knowledge, interprofessional familiarity, and a proactive approach to bridging system gaps [29]. This requires not only inter-organizational collaboration but also the meaningful engagement of patients and families.

Although the care coordinator's role is central to facilitating nursing care coordination [12, 62, 74], it is worth questioning whether a single coordinator can realistically fulfill every aspect of seamless care on their own. Our findings suggest that the care coordinator role often lacks clarity and is marked by ambiguities. Although the title would suggest a position of power and agency, in practice, coordinators frequently encounter limitations, especially when trying to bridge entrenched organizational divides within hierarchical healthcare systems. Achieving seamless care goes beyond logistical coordination. It also involves navigating the cultural differences, diverse professional languages, and contrasting philosophies of care that exist within and between healthcare organizations [75].

Although care coordinators are unquestionably valuable [30, 35, 76], perhaps it is time to reassess the scope and structure of their role. Rather than placing the responsibility of integration solely upon care coordinators, policymakers might need to look towards co-creation models of care—emphasizing shared responsibilities, fostering inter-organizational collaborations, and nurturing a culture of open dialogue. This would distribute responsibilities more evenly across inter-organizational healthcare teams, taking advantage of the strengths of various professionals and organizations [77, 78]. A shift in this direction could enhance care delivery by encouraging a system-wide transformation that transcends organizational silos, ultimately leading to a more sustainable, resilient, and integrated approach where the patient and their family is placed at the center of care [79]. Involving patients and their families as active participants in the care process aligns with concepts like patient activation and co-creation of care [80–82]. Embracing collaborative models of care could alleviate some of the pressures on individual care coordinators, fostering a more flexible healthcare system.

### Strengths and limitations

This study has several strengths, including the use of a comprehensive methodology that incorporated participant observations, interviews, and informal interviews to capture the nuances of inter-organizational collaboration. The use of multiple data sources facilitated a deep understanding of the subject and enriched the findings. The study also benefited from a strong validation process, with findings resonating with healthcare professionals during interprofessional seminars, confirming their relevance in real-world settings. Furthermore, the theory generated from this research holds the potential for broader applicability in other healthcare systems, though this remains to be explored.

However, the study also has limitations. The primary limitation is the exclusion of perspectives from patients, their families, and informal caregivers, which could improve understanding and provide additional depth to the findings. Their inclusion is recommended in future research. Moreover, data collection was conducted during a pandemic, which not only necessitated the use of video technologies like Zoom or Skype for interviews but may also have impacted the coordination of care itself. The pandemic introduced additional challenges such as disrupted workflows, increased reliance on remote communication, and reduced in-person interactions among healthcare professionals. Although video interviews have been validated as effective alternatives to face-to-face interviews [54, 83], these disruptions may have influenced the depth of interaction compared with in-person methods. Lastly, the transferability of the findings may be limited to healthcare and social care systems similar to those studied. Nevertheless, the comprehensive methodology suggests the potential for broader applicability, warranting further investigation in diverse settings.

### Implications for policy and practice

The theory "negotiating care in organizational borderlands" illustrates that inter-organizational collaboration may span from simple division, which risks creating care fragmentation, to full alliance, promoting true integration. Key strategies to promote coordination of care include fostering a more interconnected healthcare system, implementing robust communication systems, and leveraging digital tools to ensure timely and accurate sharing of patient information and knowledge across care provider boundaries. Clear definitions and understandings of professional roles and responsibilities are essential, particularly in overlapping areas, to ensure accountability and continuity in care delivery. Policies must reflect the challenges that healthcare professionals face, aligning policy design with implementation realities. By targeting interventions in organizational borderlands—where professional domains intersect—healthcare processes and outcomes can be effectively managed and enhanced, aligning care delivery with both systemic and person-centered needs.

### Future research

Although our study provides valuable insights into inter-organizational collaboration across care providers, there is scope for further exploration. Future research could examine how the positions on the spectrum—division, connection, and alliance—manifest across different patient groups. Investigating these dynamics in various clinical contexts could enhance the applicability of our grounded theory and identify unique challenges and opportunities specific to different patient populations. Additionally, exploring how communication practices are characterized within each position on the spectrum would offer deeper understanding of the mechanisms that facilitate or hinder effective collaboration. Such research could focus on the specific communication strategies employed by healthcare professionals and how these impact care coordination and patient outcomes.

## Conclusions

This study presents a grounded theory of how inter-organizational collaboration supports coordination of care for patients with complex care needs. Organizational boundaries are important and should not be removed to promote effective care coordination. Instead, the key lies in establishing effective pathways for robust inter-organizational collaboration. In a fragmented healthcare system, the ability of healthcare professionals to collaboratively bridge professional and organizational divides is fundamental, uniting their expertise to promote the experience of seamless care. This study underscores the significance of clear lines of accountability, facilitating streamlined collaborative efforts and ensuring cohesive patient care as healthcare environments evolve. The findings show that sharing responsibilities and fostering a culture of open dialogue are important in integration of care. Shared decision-making is integral, ensuring inclusion of all types of expertise and perspectives. As healthcare increasingly adopts an interdisciplinary approach, navigating organizational borderlands becomes vital for delivering coordinated care. Importantly, the seamlessness of care perceived by patients may contrast with the fragmented roles and responsibilities navigated by healthcare professionals, underscoring the complexity of their collaborative processes.

## Supplementary Information

Supplementary Material 1.

Supplementary Material 2.

## Data Availability

The datasets analyzed during the current study are available from the corresponding author on reasonable request.
