# Supplementary material for: Negotiating care in organizational borderlands: a grounded theory of inter-organizational collaboration in coordination of care
Source: BMC Health Serv Res. 2024 Nov 20;24:1438. doi: 10.1186/s12913-024-11947-4 (PMC11577764; doi:10.1186/s12913-024-11947-4)
Supplement: Supplementary file 1 — Supplementary Material 1. [file 12913_2024_11947_MOESM1_ESM.docx]

Appendix S1 – COREQ checklist

Negotiating care in organizational borderlands: a grounded theory of interprofessional collaboration in coordination of care

Consolidated criteria for reporting qualitative studies (COREQ): 32-item checklist. Developed from: Tong A, Sainsbury P, Craig J. Consolidated criteria for reporting qualitative research (COREQ): a 32 item checklist for interviews and focus groups. International Journal for Quality in Health Care. 2007. Volume 19, Number 6: pp. 349 – 357

| No Item | Guide question / description | Reported on page # |
| --- | --- | --- |
| **Domain 1: Research team**  **and reflexivity** |  |  |
| *Personal Characteristics* |  |  |
| 1. Interviewer/ Facilitator | Which author/s conducted the interview or focus group? | Performed by the first author.  See Methods, Data collection  p.11-12 and author contributions |
| 1. Credentials | What were the researcher’s credentials? E.g. PhD, MD | The researchers were four PhDs, and one PhD student  See Title page |
| 1. Occupation | What was their occupation at the time of the study? | The researchers were three PhDs working at Linnaeus University, one PhD working at University West and one PhD student at Linnaeus University, working at the ambulance service in Region Kalmar County  See affiliations |
| 1. Gender | Was the researcher male or female? | Female |
| 1. Experience and training | What experience or training did the researcher have? | The first author is a PhD student with supervision by the co-authors who had experience in this method.  Potential author bias was handled by review of memos and peer debriefing during the analysis process.  See strengths and limitations p.34-35 |
| *Relationship with participants* |  |  |
| 1. Relationship established | Was a relationship established prior to study commencement? | Researchers established contact with top managers and heads of departments. Informants did not have any relationship with the researcher prior to the study.  See Method, Recruitment of participants p.9-10 |
| 1. Participant knowledge of the interviewer | What did the participants know about the researcher? e.g. personal goals, reasons for doing the research | Informants were introduced to the research by the heads of departments and did not have contact with the researchers prior to interviews or observations. During the observations, the researcher was present and recognizable to the participants, but not an active participant with a role in the social context.  See Methods, Observations p.12 |
| 1. Interviewer characteristics | What characteristics were reported about the interviewer/facilitator? e.g. Bias, assumptions, reasons and interests in the research topic | Basic information was reported about the interviewer for the participant to get a picture of the person to meet with. The relation between researcher and research context in constructivist grounded theory is discussed in strengths and limitations p.34-35 |
| **Domain 2: study design** |  |  |
| *Theoretical framework* |  |  |
| 1. Methodological orientation and Theory | What methodological orientation was stated to underpin the study? e.g. grounded theory,  discourse analysis, ethnography, phenomenology, content analysis | Constructivist grounded theory  See Methods, Design p.7 |
| *Participant selection* |  |  |
| 1. Sampling | How were participants selected? e.g. purposive, convenience, consecutive, snowball | In accordance with constructivist grounded theory, a purposive and theoretical sampling approach was used  See Methods, Recruitment of participants p.9-10 |
| 1. Method of approach | How were participants approached? e.g. face-to-face, telephone, mail, email | See Methods, Recruitment of participants p. 9-10 |
| 1. Sample size | How many participants were in the study? | In total, 86 participants.  Methods, Recruitment of participants p. 9-10 and Table 1 p.10-11 |
| 1. Non-participation | How many people refused to participate or dropped out? Reasons? | None |
| *Setting* |  |  |
| 1. Setting of data collection | Where was the data collected? e.g. home, clinic, workplace | All observations were executed at clinic/workplace while the setting for the interviews was chosen by convenience of the participant and was thus performed via telephone or skype/zoom from home or workplace.  See Methods, Data collection p.11-13 |
| 1. Presence of non-participants | Was anyone else present besides the participants and researchers? | During the formal interviews, only the interviewer and the interviewee were present. During observations in the clinical setting, there were at times also patients and next of kin present, however they were not observed  See Methods, Data collection p.11-13 |
| 1. Description of sample | What are the important characteristics of the sample? e.g. demographic data, date | The importance of the sample is the cross-organizational representation and diversity of healthcare professionals which is reported in Methods, Recruitment of participants p.9-10 and Table 1 p.10-11 |
| *Data collection* |  |  |
| 1. Interview guide | Were questions, prompts, guides provided by the authors? Was it pilot tested? | An initial interview guide with open-ended questions were created and used. The questions were further consecutively developed in accordance with constructivist grounded theory  See Methods, Data collection p.11-13 |
| 1. Repeat interviews | Were repeat interviews carried out? If yes, how many? | No |
| 1. Audio/visual recording | Did the research use audio or visual recording to collect the data? | Yes the interviews were audio recorded, however not the observations.  See Methods, Data collection p.11-13 |
| 1. Field notes | Were field notes made during and/or after the interview or focus group? | Field notes were written adjacent to observations  See Methods, Data collection p.11-13 |
| 1. Duration | What was the duration of the interviews or focus group? | See Methods, Data collection, Observations and Interviews p.11-13 |
| 1. Data saturation | Was data saturation discussed? | Yes  Theoretical saturation according to constructivist grounded theory was attained  See Method, Recruitment of participants p.9-10 |
| 1. Transcripts returned | Were transcripts returned to participants for comment and/or correction? | Yes, no correction was needed. |
| **Domain 3: analysis and findings** |  |  |
| *Data analysis* |  |  |
| 1. Number of data coders | How many data coders coded the data? | The main author coded the data while the co-authors contributed to validation of coding  See author contributions.  See Methods, Data analysis p.9-10 |
| 1. Description of the coding tree | Did authors provide a description of the coding tree? | In part. The coding was performed by constant comparative analysis according to constructivist grounded theory  See Methods, Data analysis p.13-15. See Figure 1 p.15 |
| 1. Derivation of themes | Were themes identified in advance or derived from the data? | Derived from data as the analysis was inductive. See Methods, Data analysis p.13-15 |
| 1. Software | What software, if applicable, was used to manage the data? | Microsoft Excel  See Methods, Data analysis p.14 |
| 1. Participant checking | Did participants provide feedback on the findings? | Author bias were handled by peer debriefing and participant confirmation of the findings as the authors reiterated and confirmed emerging findings with healthcare and social care professionals outside the study. See strengths and limitations p. 34-35 |
| *Reporting* |  |  |
| 1. Quotations presented | Were participant quotations presented to illustrate the themes / findings? Was each quotation identified? e.g. participant number | Yes. Each quotation was identified with participant´s number and profession. See Findings section – for example p.19 |
| 1. Data and findings consistent | Was there consistency between the data presented and the findings? | Consistency between data and the findings is presented with quotations. Credibility is discussed in strengths and limitations p.34-35 |
| 1. Clarity of major themes | Were major themes clearly presented in the findings? | Yes. See Findings section p.16  And Figure 2 p.18 |
| 1. Clarity of minor themes | Is there a description of diverse cases or discussion of minor themes? | Yes  See Findings section p.16-27 along with Figure 2. |
