# Supplementary material for: Negotiating care in organizational borderlands: a grounded theory of inter-organizational collaboration in coordination of care
Source: BMC Health Serv Res. 2024 Nov 20;24:1438. doi: 10.1186/s12913-024-11947-4 (PMC11577764; doi:10.1186/s12913-024-11947-4)
Supplement: Supplementary file 2 — Supplementary Material 2. [file 12913_2024_11947_MOESM2_ESM.docx]

# Appendix S2 - Interview guide (translated from Swedish)

## Introduction

1. **Role and function**
   - Describe your role and function in the care chain for patients with complex care needs. What does your work entail?
2. **Understanding collaboration**
   - Can you describe what interprofessional collaboration in coordinating care means to you? How can it be created in everyday healthcare practice?

## Successful collaboration

1. **Examples of success**
   - Can you describe a successful collaboration regarding a patient? What made it successful?
   - How did different team members contribute to the success?
2. **Coordination practices**
   - What specific practices or strategies were used to ensure effective collaboration and coordination of care?
   - How was communication managed among the different professionals involved?

## Challenges and improvement

1. **Challenges in collaboration**
   - What are the main challenges you face in collaborating with other professionals to coordinate care for patients with complex needs?
   - Can you provide an example of a situation where collaboration did not work well? What were the consequences?
2. **Improvement areas**
   - Based on your experience, what could be improved in the current system to facilitate better interprofessional collaboration?
   - What changes do you see as necessary to enhance coordination of care?

## Coordination strategies

1. **Coordination techniques**
   - How do you coordinate care with other professions or care providers for patients with complex needs? What techniques or tools do you use?
   - What professions or care providers do you most frequently interact with, and how do you ensure smooth coordination?
2. **Holistic view**
   - Who in your team or organization has a holistic understanding of a patient’s situation, and how do they contribute to the care process?
   - How do you ensure that the patient's holistic needs are met?

## Conclusion

1. **Key factors for success**
   - What are the most important factors that contribute to effective interprofessional collaboration and coordination of care?
   - How could the current system be changed to better support these factors?
2. **Final thoughts**
   - Is there anything you would like to add about interprofessional collaboration and coordination of care for patients with complex needs?
